# Quantitative pupillometry for the monitoring of intracranial hypertension in patients with severe traumatic brain injury

**DOI:** 10.1186/s13054-019-2436-3

**Published:** 2019-05-02

**Authors:** Fritz-Patrick Jahns, John Paul Miroz, Mahmoud Messerer, Roy T. Daniel, Fabio Silvio Taccone, Philippe Eckert, Mauro Oddo

**Affiliations:** 1 Department of Intensive Care Medicine, Centre Hospitalier Universitaire Vaudois (CHUV), Faculty of Biology and Medicine, University of Lausanne, Rue du Bugnon 46, BH 08.623, CH-1011 Lausanne, Switzerland; 2 Critical Care Clinical Research Unit, Centre Hospitalier Universitaire Vaudois (CHUV), Faculty of Biology and Medicine, University of Lausanne, 1011 Lausanne, Switzerland; 3 Department of Clinical Neurosciences, Neurosurgery Service, Centre Hospitalier Universitaire Vaudois (CHUV), Faculty of Biology and Medicine, Lausanne University Hospital, 1011 Lausanne, Switzerland; 4 Department of Intensive Care Medicine, Erasme University Hospital, Brussels, Belgium

**Keywords:** Traumatic brain injury, Pupillometry, Pupillary reactivity, Neurological Pupil index, Intracranial pressure, Intracranial hypertension, Outcome, Prognosis

## Abstract

**Background:**

Elevated intracranial pressure (ICP) is frequent after traumatic brain injury (TBI) and may cause abnormal pupillary reactivity, which in turn is associated with a worse prognosis. Using automated infrared pupillometry, we examined the relationship between the Neurological Pupil index (NPi) and invasive ICP in patients with severe TBI.

**Methods:**

This was an observational cohort of consecutive subjects with severe TBI (Glasgow Coma Scale [GCS] < 9 with abnormal lesions on head CT) who underwent parenchymal ICP monitoring and repeated NPi assessment with the NPi-200® pupillometer. We examined NPi trends over time (four consecutive measurements over intervals of 6 h) prior to sustained elevated ICP > 20 mmHg. We further analyzed the relationship of cumulative abnormal NPi burden (%NPi values < 3 during total ICP monitoring time) with intracranial hypertension (ICHT)—categorized as refractory (ICHT-r; requiring surgical decompression) vs. non-refractory (ICHT-nr; responsive to medical therapy)—and with the 6-month Glasgow Outcome Score (GOS).

**Results:**

A total of 54 patients were studied (mean age 54 ± 21 years, 74% with focal injuries on CT), of whom 32 (59%) had ICHT. Among subjects with ICHT, episodes of sustained elevated ICP (*n* = 43, 172 matched ICP-NPi samples; baseline ICP [T_− 6 h_] 14 ± 5 mmHg vs. ICPmax [T_0 h_] 30 ± 9 mmHg) were associated with a concomitant decrease of the NPi (baseline 4.2 ± 0.5 vs. 2.8 ± 1.6, *p* < 0.0001 ANOVA for repeated measures). Abnormal NPi values were more frequent in patients with ICHT-r (*n* = 17; 38 [3–96]% of monitored time vs. 1 [0–9]% in patients with ICHT-nr [*n* = 15] and 0.5 [0–10]% in those without ICHT [*n* = 22]; *p* = 0.007) and were associated with an unfavorable 6-month outcome (15 [1–80]% in GOS 1–3 vs. 0 [0–7]% in GOS 4–5 patients; *p* = 0.002).

**Conclusions:**

In a selected cohort of severe TBI patients with abnormal head CT lesions and predominantly focal cerebral injury, elevated ICP episodes correlated with a concomitant decrease of NPi. Sustained abnormal NPi was in turn associated with a more complicated ICP course and worse outcome.

## Background

Traumatic brain injury (TBI) is a leading cause of death and disability worldwide and accounts for around 30% of all injury-related deaths [1]. Following the primary cerebral insult, TBI management focuses on the prevention and treatment of secondary brain damage, including intracranial pressure (ICP) control [2]. Pupillary examination, and in particular pupillary light reactivity, plays a fundamental role in this setting and has both diagnostic and prognostic value [3]. Elevated ICP may alter brainstem function and cause abnormalities in pupil size, symmetry, and pupillary light reactivity [4–7]. Monitoring of pupillary function provides information about secondary insults (e.g., high ICP), and sustained or newfound pupillary abnormalities are associated with a worse outcome [8]. Pupillary light reactivity is a validated predictor in both the CRASH (Corticosteroid Randomization after Significant Head Injury) and IMPACT (International Mission for Prognosis and Analysis of Clinical Trials) TBI prognostic models [9]. In the current clinical practice however, pupillary examination is generally performed using a manual, hand-held light source (e.g., pen torch); therefore, the evaluation of pupillary size and reactivity is essentially based on a visual qualitative assessment (i.e., absent or present, slow or brisk). This subjective, qualitative method has various limitations, including limited precision (especially in patients with small pupil size) and significant intra- and inter-observer variability, due to the inconsistent experience and visual acuity of the assessing clinician, differences in ambient light exposure between measurements, or the technique used to direct the stimulus (i.e., intensity, proximity, duration, and orientation of the light source) [10–12].

The use of quantitative, infrared technology for pupillary examination has long been described in ophthalmology and anesthesiology research [6, 13, 14]. While interest in its role in neurocritical care has progressively grown in recent years [7], few studies have been performed to date to determine its potential use as a technique of non-invasive neuro-monitoring follow-up in critically ill patients. An observational study in patients with normal ICP showed that standard measurements of pupillary function may be inaccurate in correctly detecting the strength of pupillary light reactivity when compared to quantitative, automated infrared pupillometers [10]. Emerging data also suggest that quantitative pupillometry may be helpful in detecting intracranial midline shift [15] and in monitoring the effects of ICP osmotic therapy on pupillary function [16].

The main objective of this study was to examine the association between elevated ICP and quantitative pupillometry-derived data in comatose patients with severe TBI and invasive parenchymal ICP monitoring, at high risk of intracranial hypertension due to abnormal head CT scan lesions. We particularly focused on the trends over time of the Neurological Pupil index (NPi) prior to sustained elevated ICP. We further investigated whether sustained abnormalities of the NPi during intracranial monitoring were associated with a more complicated ICP course and with a worse 6-month neurological outcome.

## Methods

### Study population and design

An observational cohort study was performed between November 2016 and May 2018 at the Department of Adult Intensive Care Medicine, Lausanne University Hospital (Centre Hospitalier Universitaire Vaudois, CHUV), in Lausanne, Switzerland. Subjects were patients with severe TBI (defined by a post-resuscitation Glasgow Coma Scale < 9) with abnormal head CT lesions (intracranial contusions, subdural hematoma), who underwent invasive ICP monitoring (using a parenchymal Codman ICP probe®; Codman, Raynham, MS, USA) and repeated quantitative NPi assessment (using the NPi-200 pupillometer®, Neuroptics, Laguna Hills, CA, USA) as part of routine care. Patients were excluded from the present analysis if they had visible, direct compression of the optic tracts on imaging; had previous known ophthalmologic conditions (including cataract surgery); died within 24 h of admission; or had incomplete pupillometry data. The study was approved by the ethical committee of the University of Lausanne, with a waiver of consent provided, given the retrospective observational design. Reporting of the study conforms to the STROBE statement for the report of observational cohort studies.

### General patient management

Patients were treated according to a standard protocol for the management of severe TBI, in line with the current recommended guidelines [17]*.* All patients were sedated (with propofol, at a maximal dose of 4 mg/kg/h, and sufentanil, at a maximal dose of 20 μg/h) and mechanically ventilated, aiming to keep PaO_2_ and PaCO_2_ between 90 and 100 mmHg and 36 and 40 mmHg, respectively. Cerebral perfusion pressure was maintained between 60 and 70 mmHg, with the use of isotonic fluids (aiming for normovolemia) and vasopressors (norepinephrine). Metabolic control included the maintenance of normoglycemia (arterial blood glucose between 6 and 8 mmol/L, with the use of a continuous insulin infusion) and normothermia (core body temperature < 37.8 °C) and early institution of enteral nutrition.

### Management of intracranial hypertension

Treatment of elevated ICP episodes (ICP > 20 mmHg for > 10 min) followed a stepwise management algorithm. Medical management consisted of deep sedation (with temporary increases in the infusion rates and boluses of propofol ± midazolam, aiming for a Richmond Agitation-Sedation Scale of − 4 to − 5), moderate hyperventilation (PaCO_2_ 30–35 mmHg), controlled normothermia (35–37 °C, targeted to ICP control), and osmotherapy, consisting of intravenous boluses (over 20 min) of 7.5% hypertonic saline (2 mL/kg) or 20% mannitol (0.5 g/kg). If elevated ICP levels were subsequently controlled, sedation doses were readjusted to previous levels. Therapeutic hypothermia (< 35 °C) and barbiturate coma were not part of the standard management algorithm.

### Quantitative pupillometry

The NPi®-200 pupillometer (Neuroptics, Laguna Hills, CA, USA) is a non-invasive device that uses an infrared camera that integrates a calibrated light stimulation of fixed intensity (1000 lux) and duration (3.2 s), allowing for a rapid and precise measure (0.05 mm limit) of the pupil size and of a series of dynamic pupillary variables (including the percentage pupillary constriction, latency, constriction velocity, and dilation velocity). Based on the integration of these variables into an algorithm, the NPi®-200 pupillometer calculates the Neurological Pupil index (NPi), a proprietary scalar index with values between 0 and 5 (with a 0.1 decimal precision) [4]. Pathological NPi values were defined as < 3, in line with previous reports [4, 5, 18]. Pupillometry readings were taken from every patient admitted to the unit with a TBI, at an interval of about 2 h, as part of standard of care. Ambient light (entering the non-measured pupil) was approximately the same during each NPi measurement.

### Data collection

Demographic variables included age, gender, post-resuscitation GCS, Marshall CT classification score, and type of injury (focal/unilateral vs. diffuse/bilateral). Intracranial hypertension was categorized as non-refractory (i.e., responsive to medical management, including osmotherapy, with ICP returning to < 20 mmHg) or refractory (persistent, sustained ICP elevation > 25 mmHg requiring surgical decompression). Neurological outcome was assessed by face-to-face interview at 6 months, using the Glasgow Outcome Score (GOS), categorized as poor (GOS 1–3) and good (GOS 4–5) [18].

### Data processing

For each patient, we first identified episodes of sustained ICP > 20 mmHg, and then matched ICP values to concomitant NPi measurements. For the purpose of this study, we aimed to obtain four consecutive NPi values during the 6 h prior to the maximum ICP, i.e., a baseline at − 6 h, two intermediate time points (*T*_i_ and *T*_ii_, corresponding to − 4 h and − 2 h, respectively), and the NPi min at the time of maximum ICP. NPi values used for the analysis were those of the lowest NPi value between both eyes; in cases where the NPi was abnormal on one side but normal on the other, the lowest value was considered for analysis. For ICP values, the time points corresponded to baseline ICP, *T*_i_, *T*_ii_, and ICP max. For each patient, a maximum of three separate episodes of raised ICP were included in the analysis.

Using the same approach, we also examined the NPi trends following osmotherapy (i.e., four repeated NPi values during the 6 h following treatment of elevated ICP with mannitol or hypertonic saline).

For each patient, we also calculated the cumulative abnormal NPi burden, as the number of measurements with abnormal NPi values (< 3) divided by the total number of NPi measurements during the entire ICP monitoring time. We further categorized patients according to their NPi trends following NPi abnormalities, dichotomized as no NPi recovery (patients in whom there was no recovery of normal NPi values following abnormal NPi) vs. NPi recovery (patients in whom the NPi recovered to normal values following abnormal NPi).

### Statistical analysis

Data are presented as mean (± standard deviation) or median (interquartile range, IQR) values, as appropriate according to data distribution. For the analysis of the NPi trends over time during elevated ICP episodes, comparisons were analyzed using ANOVA for repeated measures; when the effect of time was significant (*p* < 0.05), pairwise comparisons of baseline NPi with consecutive NPi values during the episode of elevated ICP (at three time points: *T*_i_, *T*_ii_, NPi min) were performed with Fisher least significant difference. The same approach was used for the analysis of the NPi trends following ICP osmotic therapy. Associations of the cumulative abnormal NPi burden with outcome variables (including intracranial hypertension and 6-month GOS) were analyzed with a non-parametric Wilcoxon test. Statistical analysis was performed with the JMP-12® package software (SAS Institute, NC, USA). Statistical significance was set at *p* < 0.05.

## Results

**Fig. 1:**
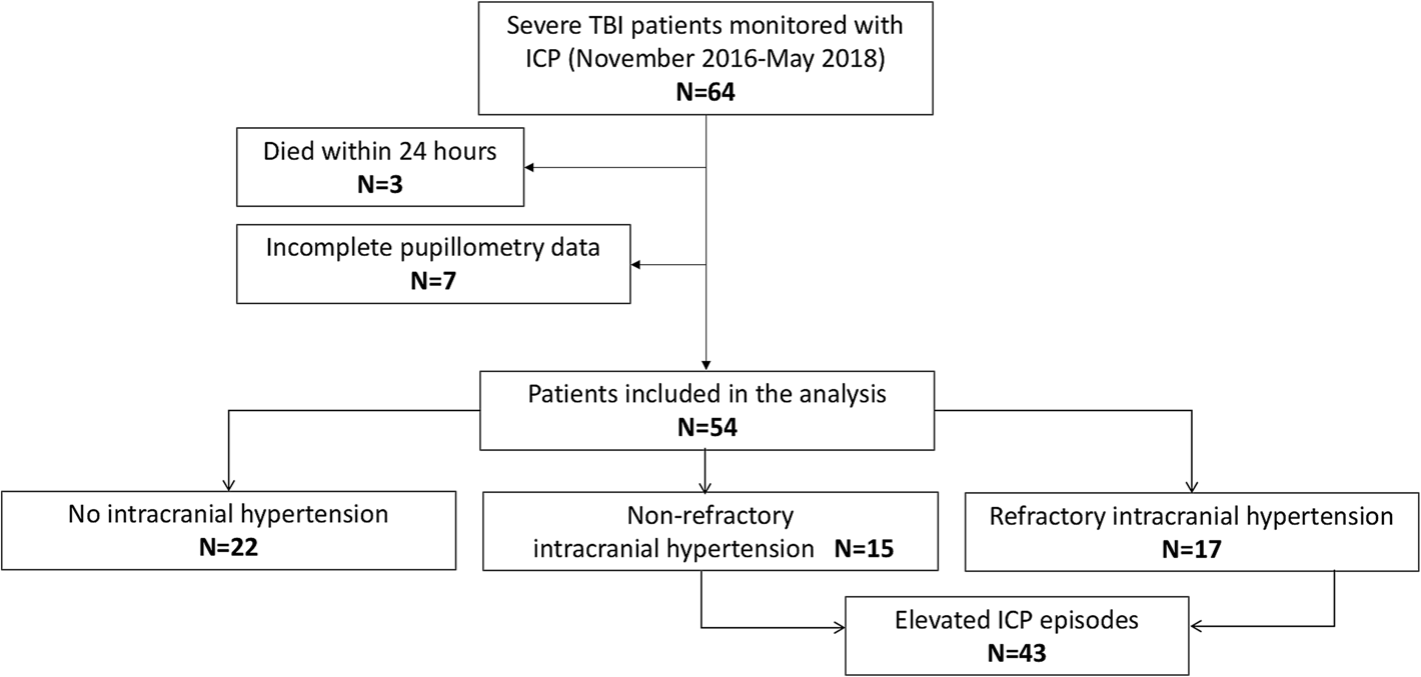
Study flow chart

A total of 54 patients were studied (Fig. 1), who underwent a median of 78 pupillometry measurements (IQR 47–108) during the monitoring time. No patient was excluded because of a previously known ophthalmologic condition or due to compression of the optic tracts. Table 1 illustrates patient demographics and neurological outcome at 6 months. The studied cohort had a relatively advanced age (average 54 years), had a high Marshall head CT score (mean 4), predominantly consisted of focal injury TBI subtype (74% of patients; mainly subdural hematoma), and had a high prevalence (59%) of intracranial hypertension, thereby explaining the elevated mortality (37%). The observed mortality in this study was actually lower than expected according to the CRASH TBI prognostic model (average predicted mortality 50%) [9].

**Table 1:** Patient demographics and outcome

Variable	Value
Total patient number	54
Age, years	54 ± 21
Female gender, *n* (%)	15 (28)
Post-resuscitation Glasgow Coma Scale	6 ± 2
Marshall head CT score	4 ± 1
CT scan injury type, *n* (%)	
Diffuse/bilateral injury	14 (26)
Focal/unilateral injury	40 (74)
Intracranial hypertension, *n* (%)	
No intracranial hypertension	22 (41)
Non-refractory intracranial hypertension*	15 (28)
Refractory intracranial hypertension**	17 (31)
6-month Glasgow Outcome Score (GOS), *n*	
Good outcome	
GOS 5 (full recovery)	5
GOS 4 (moderate disability)	13
Poor outcome	
GOS 3 (severe disability)	13
GOS 2 (vegetative state)	0
GOS 1 (death)^#^	18

Data are presented as mean ± standard deviation, unless otherwise stated

*Responding to medical management including osmotherapy

**Refractory to medical management, requiring surgical decompression

^#^Cause of death: withdrawal of life support (*n* = 16), brain death (*n* = 2)

### The relationship between invasive ICP and non-invasive NPi

**Fig. 2:**
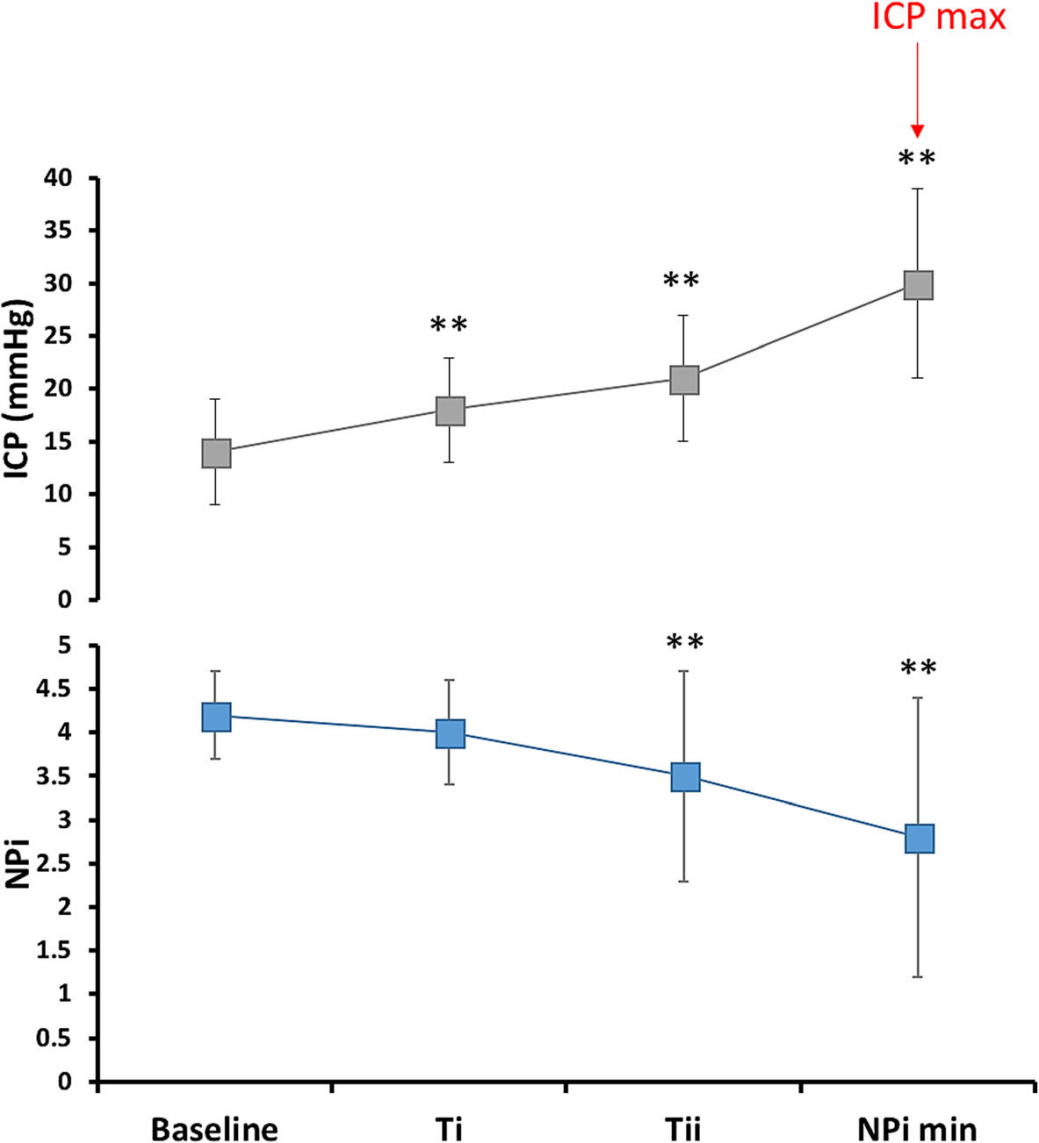
Trends over time of the Neurological Pupil index (NPi) during episodes of sustained elevated intracranial pressure (ICP). Line graphs illustrating trends over time of the NPi (blue line) during 43 episodes of elevated ICP (gray line). Data are mean ± standard deviation of a total of 172 paired ICP-NPi measurements; **p < 0.001 for pairwise comparisons of baseline ICP and NPi values (6 h previous to ICP max [red arrow]) with *T*_i_ (≈ − 4 h), *T*_ii_ (≈ − 2 h), ICP max, and NPi min, respectively (time 0)

A total of 43 episodes of sustained, elevated ICP were available for analysis. Figure 2 illustrates the trends over time of the NPi (blue squares) during elevated ICP (gray squares). ICP increased from a baseline 14 ± 5 mmHg vs. 18 ± 5 mmHg (*T*_i_), 21 ± 6 mmHg (*T*_ii_), and 30 ± 9 mmHg (ICP max; all *p* < 0.001 compared to baseline, ANOVA for repeated measures). ICP increase was associated with a concomitant and clinically relevant decrease in NPi from a baseline of 4.2 ± 0.5 vs. 4 ± 0.6 at *T*_i_ (*p* = 0.14), 3.5 ± 1.2 at *T*_ii_ (*p* < 0.0001), and 2.8 ± 1.6 at NPi min (*p* < 0.0001).

**Fig. 3:**
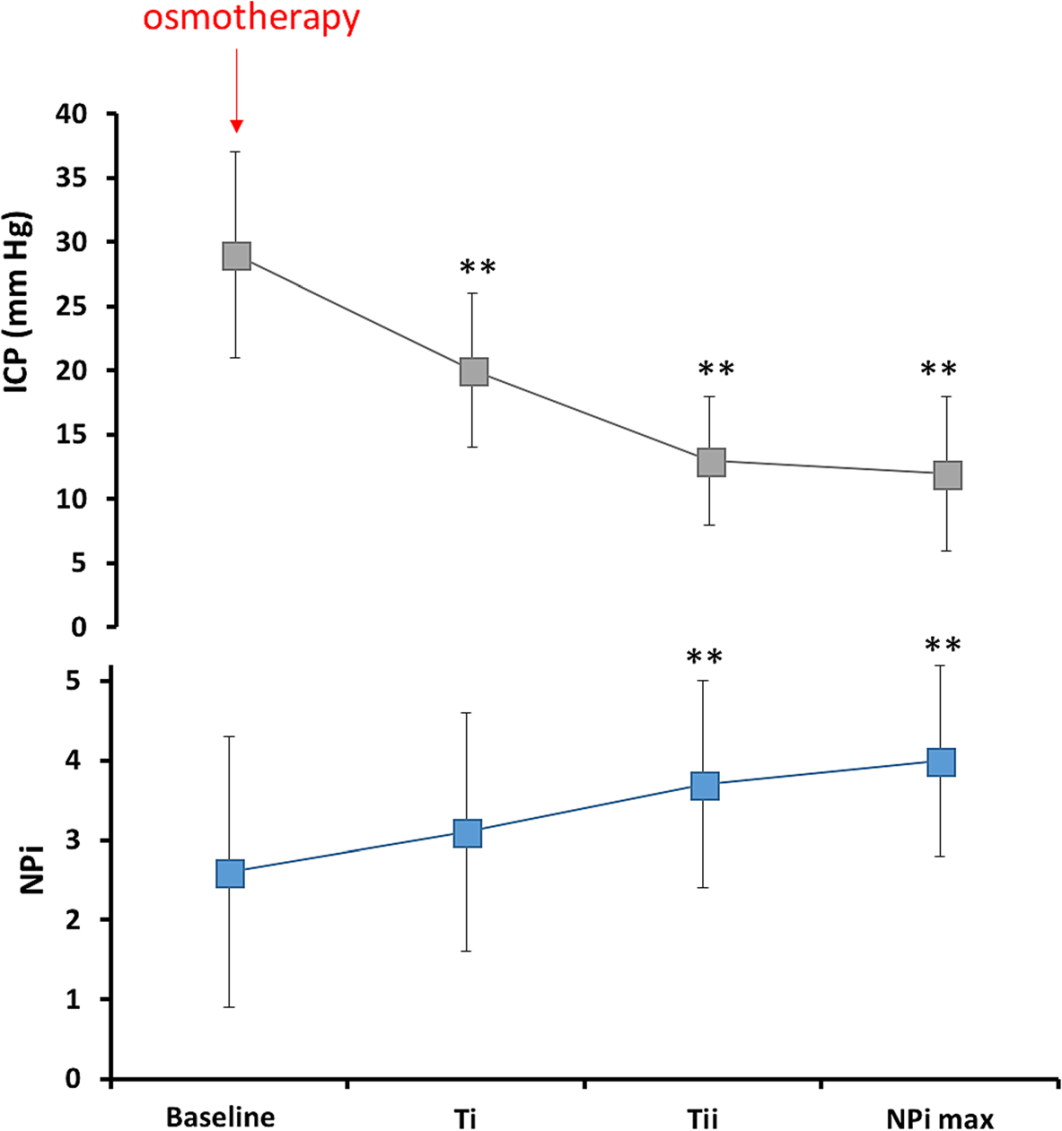
Trends over time of the Neurological Pupil index (NPi) during elevated intracranial pressure (ICP) treated with osmotherapy (mannitol or hypertonic saline bolus). Line graphs illustrating trends over time of the NPi (blue line) during 15 episodes of elevated ICP (gray line). Data are mean ± standard deviation of a total of 50 paired ICP-NPi measurements; **p < 0.001 for pairwise comparisons of baseline ICP and NPi values (start of osmotherapy [red arrow]) with *T*_i_ (≈ − 4 h), *T*_ii_ (≈ − 2 h), ICP min, and NPi max, respectively (time 0)

We further examined 15 episodes wherein osmotherapy (with either mannitol or hypertonic saline) was given to treat elevated ICP (Fig. 3). Baseline ICP (beginning of osmotherapy) decreased from 29 ± 8 to 12 ± 6 mmHg (ICP min; *p* < 0.0001), which was associated with a concomitant increase of baseline NPi from 2.6 ± 1.7 vs. 3.1 ± 1.5 (*T*_i_; *p* = 0.07), 3.7 ± 1.3 (*T*_ii_; *p* = 0.0006), and 4 ± 1.2 (NPi max; *p* < 0.0001).

### Abnormal Neurological Pupil index (NPi < 3) is associated with the severity of intracranial hypertension and with a worse 6-month outcome

**Fig. 4:**
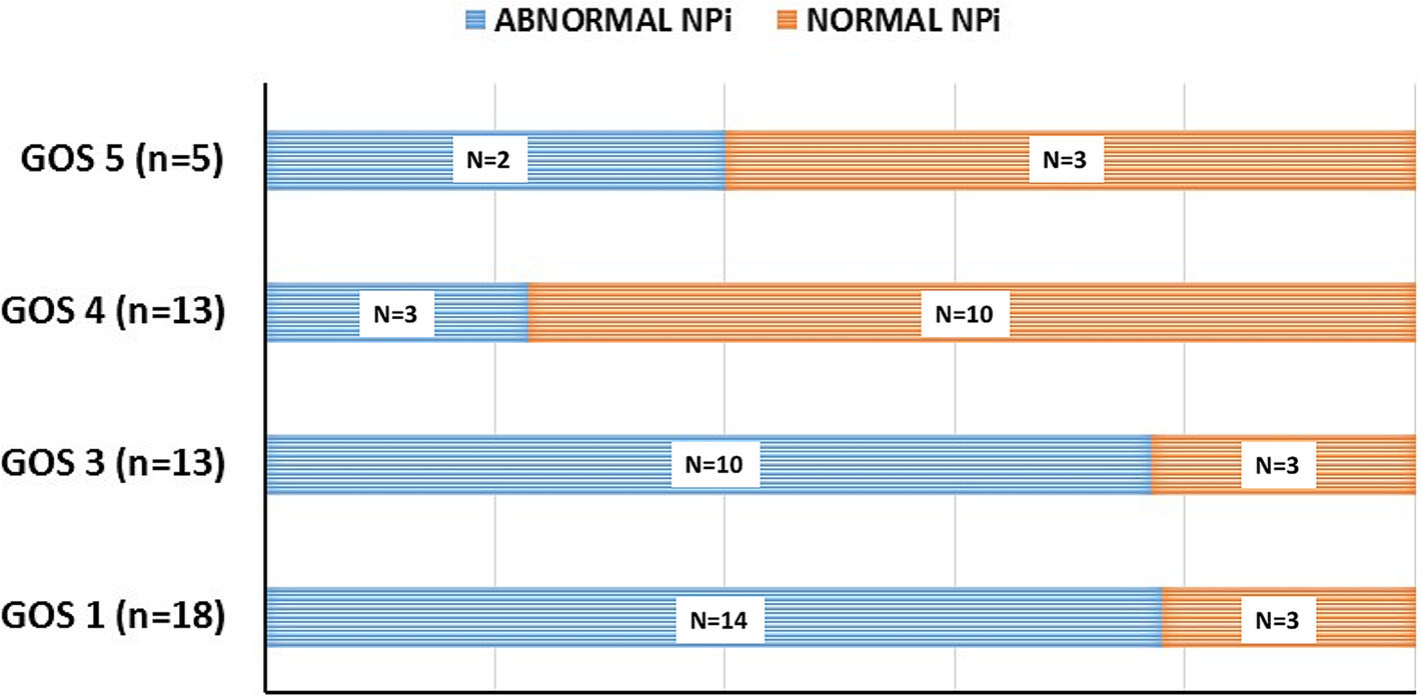
Patient distribution of abnormal vs. normal NPi across the different Glasgow Outcome Score (GOS) categories. Histograms showing that the proportion of patients with abnormal NPi was higher in patients with GOS 1 and GOS 3, as compared to patients with GOS 4 and 5

The percentage of samples with abnormal NPi values (< 3) during ICP monitoring was significantly greater in patients with refractory intracranial hypertension (38 [3–96]%) than in those with non-refractory intracranial hypertension (1 [0–9]%) or no intracranial hypertension (0.5 [0–11]%) (*p* = 0.007; Table 2). When looking at 6-month outcomes (*n* = 49; 5 patients lost to 6-month follow-up), the cumulative burden of abnormal NPi was higher in patients with a poor neurological outcome (GOS 1–3; 15 [1–80]%) when compared to those with a good outcome (GOS 4–5; 0 [0–7], *p* = 0.002; Table 2). The distribution of patients with abnormal vs. normal NPi across the different GOS categories is illustrated in Fig. 4, showing that the proportion of patients with abnormal NPi was higher in patients with GOS 1 and GOS 3 as compared to patients with GOS 4 and 5. Of note, the median number of NPi measurements during ICP monitoring per patient did not differ significantly between the poor outcome group (GOS 1 and 3; *n* = 76 [48–114]) and the good outcome group (GOS 4 and 5; *n* = 56 [31–56], *p* = 0.40), as well as the duration of ICP monitoring (7 [4–13] vs. 6 [4–9] days, respectively, *p* = 0.48).

**Table 2:** Cumulative burden of abnormal Neurological Pupil index (NPi < 3) is associated with the severity of intracranial hypertension and 6-month Glasgow Outcome Score (GOS)

Intracranial hypertension (ICHT)	No ICHT (*N* = 22)	Non-refractory ICHT (*N* = 15)	Refractory ICHT (*N* = 17)	*p* value
% measurements with abnormal NPi < 3	0.5 (0–11)	1 (0–9)	38 (3–96)	0.007*
6-month Glasgow Outcome Score (GOS)**	GOS 4–5 (*N* = 18)	GOS 1–3 (*N* = 31)		*p* value
% measurements with abnormal NPi < 3	0 (0–7)	15 (1–80)		0.002

Data are presented as median (interquartile range) number of samples with abnormal NPi < 3 during ICP monitoring time

*p value for comparison with refractory ICHT

**Five patients lost to follow-up

No recovery from an abnormal NPi was observed only in patients with intracranial hypertension (12/32; 37.5%) and was a sign of dismal prognosis (all 12 patients with no NPi recovery had a GOS 1–3 at 6 months). In patients who had a decompressive hemi-craniectomy, good 6-month outcome (GOS 4–5) was observed only in patients who had NPi recovery (6/8 patients vs. 0/9 patients with no NPi recovery; *p* = 0.0022).

Differences in the NPi values between the two eyes were frequent, with the lowest NPi value being ipsi-lateral to focal injury in 62% of cases.

## Discussion

The results of this study can be summarized as follows: (1) sustained elevations of ICP > 20 mmHg are associated with a concomitant and clinically relevant decrease of quantitative NPi, on average below normal values (NPi < 3); (2) treatment of elevated ICP with hyperosmolar agents (mannitol or hypertonic saline boluses) was in turn associated with a normalization of the NPi; (3) the cumulative burden of abnormal NPi was a marker of an increased severity of intracranial hypertension, a more complicated ICP course (requiring decompressive craniectomy), and a worse 6-month neurological outcome; and (4) failure of the NPi to recover to normal values was associated with very poor prognosis. Altogether, our data suggests that in patients with severe TBI and abnormal head CT lesions, monitoring NPi trends may be a valuable complement to invasive ICP monitoring, by providing important diagnostic and prognostic information.

Abnormal pupillary reactivity is often used in clinical decision-making to predict intracranial hypertension in severe TBI [19, 20]. Monitoring of pupillary function helps provide insight into secondary insults (e.g., high ICP), as sustained or new-onset pupillary abnormalities are associated with worse outcome [8]. Pupillary light reactivity is a known prognostic predictor in patients with TBI [21] and is a validated variable used in both the CRASH (Corticosteroid Randomization after Significant Head Injury) and the IMPACT (International Mission of Prognosis and Analysis of Clinical Trials) prognostic models [9]. Somewhat surprisingly, previous studies and both the CRASH and IMPACT models are based on the standard manual qualitative pupillary examination, and there are only limited data regarding the potential value of quantitative pupillometry in the monitoring of TBI complications and outcome. The majority of pupillometry studies was single-centered, included a relatively small sample size, and focused exclusively on the pupillary size, percentage pupillary constriction, and constriction velocity [15, 22, 23]. Two studies examined the relationship between elevated ICP and the NPi [4], as well as the dynamic response of the NPi to ICP therapy with osmotic agents [16]. Both studies had larger sample sizes but analyzed a heterogeneous group of acute brain injury patients (including TBI, intracerebral hemorrhage, and subarachnoid hemorrhage). In our study, we aimed at analyzing a homogeneous group of severe TBI patients with abnormal intracranial lesions and a high Marshall head CT score (average 4) on admission, who were at high risk of intracranial hypertension (59% prevalence). When matching the NPi values to invasive ICP during episodes of sustained elevated ICP, we found a significant inverse relationship between the two variables, both during ICP spikes and following ICP reduction with osmotherapy (mannitol or hypertonic saline boluses). An important and novel finding of our study is the association of the NPi with the severity of intracranial hypertension. For this, we first calculated the total cumulative burden of abnormal NPi (defined as NPi below 3) during ICP monitoring time and categorized TBI subjects into three subgroups, according to the severity of intracranial hypertension. The severity of intracranial hypertension was defined by the necessity of surgical decompression (*refractory* intracranial hypertension) vs. ICP medical management only (including osmotherapy; *non-refractory* intracranial hypertension) vs. no elevated ICP. Using this approach, we found that the subgroup of patients with refractory intracranial hypertension had a much greater number of abnormal NPi readings, when compared to those with non-refractory intracranial hypertension and normal ICP.

The NPi appears a valuable prognostic tool, given the observed association between cumulative NPi abnormalities and 6-month neurological outcome. In this respect, the within-patient evolution over time of the NPi seems to provide important prognostic information in the setting of post-traumatic intracranial hypertension. Indeed, absent recovery from an abnormal NPi and a trend of persistently low NPi (e.g., following decompressive craniectomy) was associated with a very poor prognosis, while on the other hand NPi recovery and improvement was a marker of a better outcome. These data appear in line with previous reports that found a relationship between patient prognosis and the response to ICP therapy [24] as well as the dose of intracranial hypertension [25]. From the pathophysiological standpoint, it also implies that NPi decrease upon elevated ICP may have two main different causes. First, intracranial hypertension may augment cerebral spinal fluid pressure around the optic nerve sheath and reduce optic nerve perfusion [26], thereby explaining the decrease of NPi associated with elevated ICP, but without necessarily implying a severe or widespread brainstem lesion or dysfunction. In this case, NPi recovery (such as after osmotherapy) may be associated with a more favorable ICP course and outcome, in line with previous reports showing that the response to ICP therapy is a major outcome determinant [24]. Second, NPi decrease may be due to direct brainstem compression or, even more likely, brain distortion due to mass effect [27], which therefore remains permanent (no NPi recovery) and would be associated with a more complicated ICP course and a worse outcome.

Altogether, our study reinforces the importance of using invasive ICP monitoring, but further suggests that the information derived from combined NPi-ICP monitoring may provide more information than ICP monitoring alone, indeed underlying the role of multimodal monitoring in patients with severe brain injury and the potential role of automated infrared pupillometry in this setting.

The potential effect of sedatives and analgesics on pupillometry values warrants further discussion. We used the NPi as the main endpoint because it appears to be less affected by sedatives and opioids in comparison to other pupillometry variables, such as the pupil size and percentage constriction. The NPi may be potentially reduced by the combined effect of high-dose remifentanil with hypercarbia and hypoxia [28]: the latter conditions did not occur during NPi measure and, although the opioid agent used for analgesia management consisted of sufentanil and not remifentanil, we did not use high-dose opioids. High-dose propofol [29] also may potentially reduce the NPi; however, the infusion dose of propofol was kept < 4 mg/kg in our study. It was shown by Delfino et al. that infused propofol at 100 μg/kg/min (= 6 mg/kg/h) resulted in bispectral index (BIS) monitoring values of approximately 42 [30], and indeed, at this infusion rate, propofol has no effect on the NPi (while it significantly reduced pupil size and percentage constriction) [31]. Finally, it is worth noting that the NPi value is adjusted to individual resting pupil size [32]. In summary, we may reasonably conclude that the combination of propofol and opioids was unlikely to produce the changes of the NPi observed during ICP episodes.

### Study limitations

The study was single-centered and included a relatively limited sample size of patients with severe TBI monitored with ICP who were at high risk for intracranial hypertension. TBI injury subtype was also predominantly focal and included a cohort with a relatively advanced age, thus limiting the generalizability of our findings. However, the inclusion of a selected and homogeneous TBI cohort also has advantages, as it identifies a potential group of severely head-injured patients, in whom (1) the addition of the NPi monitoring could be of particular value and may be helpful for individualized ICP care and (2) future larger multi-centered confirmatory studies using combined ICP and NPi monitoring may be warranted. Additional studies also may help to better refine the role of the NPi as a monitoring tool, its place in ICP management algorithms, and potential role in future guidelines for TBI care. Episodes of sustained elevated ICP were retained for the analysis based on three main criteria: (a) ICP max > 20 mmHg for at least 10 min, (b) at least three repeated consecutive NPi measurements during the 6 h preceding ICP max (and respectively following ICP osmotherapy), and (c) a maximum of three episodes per patient. While this increases data quality (particularly, by avoiding skewing of data) and therefore the robustness of the statistical analysis, it may introduce selection biases. The NPi data reported during episodes of elevated ICP may not necessarily be representative of average patient NPi during the entire ICU stay. Although all patients had NPi readings taken at least every 2 h, NPi measurements were more frequent during elevated ICP episodes (at least every hour), and in this case, the lower values were considered for the matching analysis of NPi with ICP. Finally, there was no important change in the infusion rates of sedatives during analyzed ICP episodes, but additional sedative boluses were given, and therefore (albeit unlikely), we cannot completely rule out that this may have at least partly affected the NPi.

## Conclusions

In patients with severe TBI and abnormal intracranial CT lesions at risk for secondary intracranial hypertension, sustained elevated ICP is associated with impaired NPi, which in turn may recover to normal values upon ICP treatment with osmotherapy. Sustained abnormalities of the NPi were more frequently observed in patients with refractory ICP requiring decompressive hemi-craniectomy and were associated with a worse 6-month outcome. These findings suggest that adding non-invasive NPi to invasive ICP monitoring provides important supplementary diagnostic, therapeutic, and prognostic information to guide the management of severe TBI patients.
